# Safety Profile of Nutraceuticals Rich in Coumarins: An Update

**DOI:** 10.3389/fphar.2022.803338

**Published:** 2022-01-24

**Authors:** Simona Codruta Heghes, Oliviu Vostinaru, Cristina Mogosan, Doina Miere, Cristina Adela Iuga, Lorena Filip

**Affiliations:** ^1^ Department of Drug Analysis, Iuliu Hatieganu University of Medicine and Pharmacy, Cluj-Napoca, Romania; ^2^ Department of Pharmacology, Physiology and Physiopathology, Iuliu Hatieganu University of Medicine and Pharmacy, Cluj-Napoca, Romania; ^3^ Department of Bromatology, Hygiene, Nutrition, Iuliu Hatieganu University of Medicine and Pharmacy, Cluj-Napoca, Romania; ^4^ Department of Proteomics and Metabolomics, Research Center for Advanced Medicine—MedFUTURE, Iuliu Hațieganu University of Medicine and Pharmacy, Cluj-Napoca, Romania

**Keywords:** coumarins, nutraceuticals, acute toxicity, hepatotoxicity, phototoxicity

## Abstract

Coumarins are a family of benzopyrones largely distributed in the natural kingdom, being present in the seeds, fruits, flowers, or roots of various plant species. Natural coumarin compounds are found in significant concentrations in some herbs or spices used as nutraceuticals, but they are also present in cosmetics or household products, due to their pleasant odor. Therefore, an accidental exposure to high doses of coumarins, could lead to the development of harmful effects in some patients. This review summarizes the latest published data from preclinical and clinical studies with natural coumarins, focused on the investigation of general and specific toxicity, with the aim of a better understanding of the safety profile of these valuable compounds. Regulatory aspects concerning the use of natural coumarins in several world regions are also reviewed.

## Introduction

Nutraceuticals are bioactive substances which have become increasingly popular in the last 2 decades being used worldwide for health promotion and the prevention of various diseases. They are represented by numerous phytochemicals but also fatty acids, amino acids or probiotics/prebiotics and may be responsible for a variety of biological effects (Nahar et al., 2021).

In the larger category of nutraceuticals, natural coumarins play an important role, being present in high concentrations in several dietary plant species, like tonka beans (*Dipteryx odorata* (Aubl.) Forsyth f [Fabaceae]) where they have been originally discovered in 1820, or cinnamon (*Cinnamomum verum* J. Presl [Lauraceae]), but also in a variety of foodstuffs like olive and soy oils, coffee, nuts, wine, and green tea (Lončar et al., 2020).

Many natural coumarins have been successfully tested for an array of pharmacological properties like anti-inflammatory, antioxidant, antimicrobial, antidepressant, neuroprotective or antitumoral effects (Srikrishna et al., 2018). Additionally, several natural coumarins have served as scaffolds for the development of authorized drugs like warfarin, other drug candidates with different pharmacological properties being constantly developed (Bansal et al., 2013).

Although several articles and reviews focused on the presentation of important chemical and pharmacotherapeutic aspects regarding natural coumarins have been already published (Venugopala et al., 2013; Annunziata et al., 2020;Sharifi-Rad et al., 2021), the safety profile of coumarins was not thoroughly reviewed to present date. From a toxicological point of view, the presence of several natural coumarins in spices like cassia cinnamon which is widely used for preparation of pastries, cakes, or sweet biscuits, but also in cosmetics like perfumes or sunscreens, means that multiple routes of human exposure to natural coumarins have been described, with a possible oral, pulmonary, or skin absorption and subsequent development of toxic effects. Therefore, the aim of this review was to present the latest available data from preclinical and clinical studies with natural coumarins, regarding both general toxicity and specific organ toxicities, with additional mechanistic explanations, in order to increase the awareness of healthcare and food industry professionals for a safer use of these valuable compounds.

## Natural Coumarins: Types and Sources

### Structure and Classification

**FIGURE 1:**
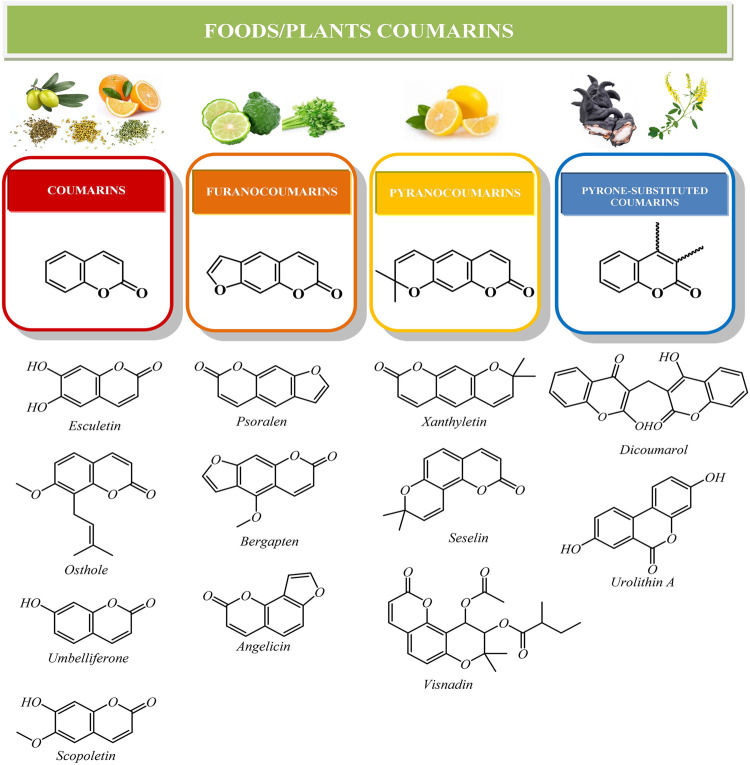
Chemical structures of the main classes of natural coumarins.

Coumarins are one of the most important classes of chemical compounds synthesized by plants, being part of the family of benzopyrones (Sarker and Nahar, 2017). Coumarin backbone (Figure 1) consists of an aromatic benzene ring in a conjugated system, which is fused with α-pyrone (lactone ring). The structure is rich in electrons and capable to react with different molecules as enzymes and receptors, which leads to potent medicinal effects (Önder, 2020).

Currently, more than 1800 coumarin-derived compounds were described (Akkol et al., 2020; Lončar et al., 2020). Naturally occurring coumarins are subdivided in different classes based on their chemical diversity and complexity: simple coumarins (esculetin, scopoletin, umbelliferone), furanocoumarins (psoralen, bergapten, angelicin), pyranocoumarins (xanthyletin, seselin) and coumarins substituted in the pyrone ring, as biscoumarins (dicoumarol) or benzocoumarins (urolithins) (Annunziata et al., 2020) (Figure 1). Additionally, simple coumarins like umbelliferone or can be linked to a C_15_ terpene moiety, forming sesquiterpene coumarins like umbelliprenin (Gliszczynska and Brodelius, 2011).

### Food and Herbal Sources of Coumarin Compounds

In nature, coumarins can be found in a free form or conjugated with other molecules like glycosides (Stringlis et al., 2019; Stassen et al., 2021). They are found in different parts of plants, such as roots, seeds, nuts, flowers and fruits of many species, being used as condiments (spices), herbal teas or medicines. In addition, coumarins can also be found in some widely used foods like oils (olive), coffee, nuts, wine, and tea (Lončar et al., 2020). Coumarins are even considered significant constituents of propolis that contribute to its pharmacological properties (esculin, daphnetin, fraxetin, umbelliferone, 4-methylumbelliferone, 4-hydroxycoumarin, scoparone, coumarin or herniarin) (Hroboňová et al., 2013).

The most significant natural coumarins in the field of phytochemistry, pharmacology, medicinal chemistry, and food science, together with their vegetal sources grouped in families are listed in Table 1.

**TABLE 1:** Main coumarins and their vegetal sources.

Plant species	Coumarins	References
Apiaceae/Umbelliferae		
Anethum graveolens (L.)	Esculetin, scopoletin, furanocoumarin, oxypeucedanin, oxypeucedanin hydrate, falcarindiol	Kovač-Bešović and Durić, (2003); Kaur and Arora, (2010)
(Dill)		
Angelica archangelica (L.)	Angelicin, osthole, bergapten, imperatorin, isoimperatorin, oreoselone, oxypeucedanin, psoralen, umbelliferone, xanthotoxin, xanthotoxol, umbelliprenin	Kumar et al., 2013; Forycka and Buchwald, (2019)
(Angelica)		
Apium graveolens (L.)	Esculetin, bergapten, celerin, celereoside, isoimperatorin, isopimpinellin, osthenol, seselin, scopoletin, psoralen, umbelliferone, xanthotoxin	Garg et al., 1979; Najda et al., 2015; Arsenov et al., 2021
(Celery)		
Coriandrum sativum (L.) (Coriander)	Coumarin 7-substituted derivatives	Sharifi-Rad et al. (2021)
Cuminum cyminum (L.) (Cumin)	Coumarin	Rebey et al. (2012)
Daucus carota (L.)	Bergapten, isopimpinellin, umbelliferone, xanthotoxin	Ozçelik and Kusmenoglu, (2004); Kenari et al., 2021
(Carrot)		
Foeniculum vulgare Mill. (Fennel)	Scopoletin, bergapten, imperatorin, 8-methoxypsoralen, psoralen	Kaur and Arora, (2010); Yang et al., 2015
Pastinaca sativa (L.)	Angelicin, bergapten, isopimpinellin, oxypeucedanin hydrate, xanthotoxin, imperatorin, psoralen	Kenari et al. (2021)
(Parsnip)		
Petroselinum crispum (Mill.) Fuss	Bergapten, oxypeucedanin, 8-metoxypsoralen, imperatorin, isoimperatorin, isopimpinellin, psoralen	Manderfeld et al., 1997; Arsenov et al., 2021
(Parsley)		
Pimpinella anisum (L.) (Aniseed)	Bergapten, scopoletin, umbelliferone, umbelliprenine	Sun et al. (2019)
Rutaceae		
Aegle marmelos (L.) Corrêa	Angelicin, umbelliferone, scopoletin, marmesinin, 8-hydroxypsoralen, marmelosin	Avula et al. (2016)
(Bael fruit)		
Citrus x aurantiifolia (Christm.) Swingle	Bergamottin, 5-geranyloxy-7-Methoxycoumarin, imperatorin, isoimperatorin, isopimpinellin, limettin, marmesin, oxypeucedanin hydrate, phellopterin, scoparone	Dugrand et al., 2013; Dugrand-Judek et al., 2015
(Lime)		
Citrus x limon (L.) Osbeck	Limettin, 5-geranyloxy-7-methoxycoumarin, oxypeucedanin hydrate, byakangelicol, oxypeucedanin, 8-geranyloxypsoralen, bergamottin, umbelliferone, heraclenin, phellopterin, osthole, auraptene, isopimpinellin, bergapten	Dugrand et al., 2013; Dugrand-Judek et al., 2015
(Lemon)		
Citrus x sinensis (L.) Osbeck	Herniarin, scopoletin, scoparone, umbelliferone, xanthyletin, bergaptol	Dugrand et al., 2013; Dugrand-Judek et al., 2015
(Sweet orange)		
Citrus x paradisi Macfad. (Grapefruit)	Bergamottin, auraptene, limettin, scopolin, bergapten, bergaptol, isopimpinellin, osthole	Dugrand et al., 2013; Dugrand-Judek et al., 2015
Asteraceae/Compositae		
Arnica montana (L.) (Arnica)	Scopoletin, umbelliferone	Kriplani et al. (2017)
Chamaemelum nobile (L.) All	Scopolin (7-β-d-glucopyranosyl-scopoletin), umbelliferone, herniarin, scopoletin	European Medicines Agency, (2011)
(Roman Chamomile)		
Cichorium intybus (L.) (Chicory)	Umbelliferon, esculetin (6,7-dihydrocumarin) scopoletin, esculetin and cichorin	Das et al., 2016; Aisa et al., 2020
Matricaria chamomilla (L.)	Umbelliferone, herniarin, skimmin, daphin, daphnetin	Petruľová-Poracká et al. (2013)
(Chamomille)		
Fabaceae/Leguminosae		
Dipteryx odorata (Aubl.) Forsyth f	Esculin, esculetin	Oliveros-Bastidas et al. (2013)
(Tonka Bean)		
Glycyrriza glabra (L.) (Liquorice)	Glycycoumarin, isoglycycoumarin, licopyranocoumarin, isotrifoliol, glycyrol, glycyrurol, licoarylcoumarin, glycyrin	Zang, (2020)
Trigonella foenum-graecum (L.) (Fenugreek)	Hymecromone, trigocoumarin, trigoforin, scopoletin	Dini and Laneri, (2021)
Moraceae		
Oleae europaea (L.)	Esculetin, scopoletin, esculin	Hashmi et al., 2015; European Medicines Agency, (2017)
(Olive)		
Ficus carica (L.)	Umbelliferone, psoralen, bergapten	Ammar et al. (2015)
(Fig)		
Araliaceae		
Eleutherococcus senticosus (Rupr. & Maxim.) Maxim	Isofraxidin	Guo et al. (2019)
(Siberian Ginseng)		
Lamiaceae/Labiadae		
Ocimum basilicum (L.)	Esculetin, esculin, coumarin, ocimarin	Zahran et al. (2020)
(Basil)		
Lauraceae		
Cinnamomum sp. (Cinnamon)	Coumarin, scopoletin	Wang et al., 2013; Ananthakrishnan et al., 2018
	*C. zeylanicum* has a low content compared with other species	
Persea americana Mill. (Avocado)	Scopoletin	Bhuyan et al. (2019)
Tiliaceae		
Tilia cordata Mill. (Linden)	Scopoletin	Arcos et al. (2006)
Urticaceae		
Urtica dioica (L.)	Umbelliferone, esculetin, scopoletin	Esposito et al., 2019; Repajić et al., 2021
(Nettle)		

## Safety Profile of Natural Coumarins

### General Toxicity of Natural Coumarins

The general toxicity of natural coumarins was evaluated preclinically in several acute, subacute, subchronic or chronic tests. For coumarin, the acute oral LD50 in mice was found to be 196–780 mg/kg bw with signs of liver toxicity (Lake, 1999). In rats, acute LD50 values were 290–680 mg/kg bw after oral administration, while in guinea pigs the acute oral LD50 was 202 mg/bw (IARC, 2000). In a subchronic study, B6C3F1 mice were treated orally with 19–300 mg/kg bw coumarin for 13 weeks. Although no clinical signs of toxicity were observed, a reduction of the mean body weight gain and a centrolobular hepatocellular hypertrophy were noted for the highest dose. In Sprague-Dawley rats treated orally with 50–500 mg/kg bw coumarin for 13 weeks, several signs of liver toxicity were observed (Lake and Grasso, 1996). In a chronic study on CD-1 mice treated orally for 2 years with a diet containing 300–3,000 ppm coumarin, no signs of clinical toxicity were observed, with NOAEL of 3,000 ppm or 280 mg/kg bw/day for male mice. However, Sprague-Dawley rats administered 333–5,000 ppm coumarin for 2 years showed signs of anemia and increase of alkaline phosphatase and glutamic-pyruvic transaminase (Carlton et al., 1996).

Osthole (7-methoxy-8-(3-methyl-2-butenyl)-2H-1-benzopyran-2-one) was another natural coumarin tested for acute or subchronic toxicity. The acute intraperitoneal LD50 in mice was 710 mg/kg bw, the clinical signs of toxicity being hyperventilation, tremor, and photophobia. In a subchronic study, osthole was administered to Wistar rats in doses of 5–50 mg/kg bw for 45 days, by oral route. The results showed pulmonary hemorrhage and mild inflammatory processes in kidneys and liver of the animals treated with higher doses of osthole (Shokoohinia et al., 2017).

The acute toxicity of esculetin (6,7-dihydroxycoumarin) was evaluated in mouse after oral and intraperitoneal administration. The results showed a low acute toxicity with an oral LD50 of over 2000 mg/kg bw, the intraperitoneal LD50 being 1,450 mg/kg bw (Tubaro et al., 1988). The subchronic or chronic toxicity of esculetin was not assessed.

Auraptene, a coumarin from *Citrus* species was tested for acute oral toxicity in rats in doses of 125–2000 mg/kg bw, not causing mortality or clinical signs of toxicity. In a subacute test, auraptene was administered to rats orally in doses of 125–250 mg/kg bw for 28 days, with no observed hematological, histopathological, or biochemical modifications (Vakili et al., 2017). The most significant aspects concerning toxicity of natural coumarins are listed in Table 2.

**TABLE 2:** Toxicological aspects concerning natural coumarins.

Type of toxicity	Type of study (preclinical model/clinical test/case report)	Findings	References
General toxicity	Acute toxicity of coumarin in mice	Oral LD50 of 196–780 mg/kg with signs of liver toxicity	Lake, (1999)
	Subchronic toxicity of coumarin in Sprague-Dawley rats	Signs of liver toxicity after 13 weeks of oral administration of doses over 50 mg/kg	Lake and Grasso, (1996)
	Acute toxicity of osthole in mice	Intraperitoneal LD50 of 710 mg/kg	Shokoohinia et al. (2017)
	Acute toxicity of esculetin in mice	Low toxicity with oral LD50 over 2000 mg/kg	Tubaro et al. (1988)
	Acute toxicity of auraptene in rats	No mortality or signs of toxicity after oral administration of 125–2000 mg/kg	Vakili et al. (2017)
Hepatotoxicity	Subchronic toxicity of coumarin in rats	Vacuolar degeneration and necrosis of hepatocytes after oral administration of 0.75% coumarin	Lake and Grasso, (1996)
	Acute hepatotoxicity of psoralen in rats and mice	Cholestatic liver injuries in rats only after oral administration of 80 mg/kg	Wang et al. (2019)
	Randomized control trial of coumarin in lymphedema	Under 1% incidence of hepatotoxicity in patients after oral administration of 400 mg coumarin for 14 months	Casley-Smith and Casley-Smith, (1995)
	Case report of cinnamon supplements toxicity	Hepatotoxicity with abdominal pain and liver enzymes elevation in a 73-year-old patient	Brancheau et al. (2015)
Dermatological toxicity	Case reports of fig toxicity	Photoallergic reaction to furanocoumarins confirmed by histopathological test	Bonamonte et al. (2010)
	Case reports of cinnamon flavored products toxicity	Contact stomatitis cause by cinnamon flavored chewing-gum	Calapai et al. (2014)
Reproductive and developmental toxicity	*In vitro* test of coumarin on zebrafish embryos	No modifications caused by coumarin in the developing larvae or their internal structures	Aspatwar et al. (2020)
	*In vivo* test of coumarin on Wistar rats	No effect of coumarin on parental fertility or development of rat pups	Api et al. (2019)

### Hepatotoxicity of Coumarins

Previous studies have shown that coumarin and especially its 3,4-epoxide intermediate can cause vacuolar degeneration, necrosis, and apoptosis of hepatocytes in the liver of rats fed with a 0.75% coumarin diet for 4 weeks. The histopathological modifications were accompanied by significant increases in serum bilirubin and alanine aminotransferase activity (Lake and Grasso, 1996).

A recent study investigated in depth the hepatotoxic potential of psoralen, a furanocoumarin from *Fructus Psoraleae* (the seed of *Cullen corylifolium* (L.) Medik [Fabaceae]) in rats and mice. The oral administration of psoralen in doses of 80 mg/kg bw in rats and 320 mg/kg in mice produced cholestatic liver injuries in rats but not in mice. In rat liver, psoralen decreased the expression of BSEP and MRP2, suggesting an inhibition of bile acid excretion and also reduced the expression of SULT2A1, an enzyme involved in the clearance of bile acids from the organism (Wang et al., 2019).

In humans, unlike in rats, the major metabolic pathway of coumarins is 7-hydroxylation, catalyzed by CYP2A6 enzyme which leads to the formation of 7-hydroxycoumarin, excreted by urine as conjugates with glucuronic acid or sulphate anion. However, in humans with genetic polymorphism of CYP2A6, with the apparition of the inactivating CYP2A6*2 allele, the 7-hydroxylation is deficient, leading to the accumulation of toxic 3,4-coumarin epoxide. Although the genetic polymorphism of the mentioned isoform of cytochrome P450 system is more frequent in Asians, affecting 20% of the population (Mizutani, 2003), its precise correlations with known cases of coumarin-induced liver toxicity were not thoroughly investigated.

In the clinical trials investigating possible beneficial effects of coumarin in lymphedema, data concerning possible hepatotoxic effects were often contradictory. Initially, in one clinical trial, two cases of hepatotoxicity were observed in 1,106 patients taking 400 mg coumarin daily for 14 months), suggesting a low incidence (below 1%) of this adverse effect (Casley-Smith and Casley-Smith, 1995). However, another smaller scale clinical trial highlighted an incidence rate of 9% of hepatotoxicity induced by coumarin use also for lymphedema treatment (Loprinzi et al., 1997). The large variations of the incidence of coumarin-induced hepatotoxicity observed in clinical trials are not fully understood, but they can be partially explained by differences in study designs and protocols for quantifying and interpreting the side effects.

Only a few reports signaled the apparition of hepatotoxicity in patients taking coumarin rich foods or dietary supplements. A case report study showed that cinnamon supplements used for a week by a 73-year-old patient caused a hepatitis-like syndrome with abdominal pain and liver enzymes elevation, the causality relation between the adverse effect and the ingested drug/supplement being confirmed by Naranjo algorithm (Brancheau et al., 2015). However, other molecules present in the chemical composition of cinnamon could also contribute to the hepatotoxic effect.

### Anticoagulant Effect and Risk of Hemorrhage

The anticoagulant effect of natural coumarins was firstly noticed in the first decades of the 20th century when cattle feeding on molded sweet clover (*Melilotus* spp.) died of severe hemorrhage. An investigation found that in sweet clover infected with specific fungi (*Aspergillus* spp., *Penicilium* spp.), the naturally present coumarin was converted by the fungi into 4-hydroxycoumarin which can spontaneously form dicoumarol, a potent anticoagulant which inhibits hepatic synthesis of several coagulation factors, acting as a vitamin K antagonist (Yarnell and Abascal, 2009).

However, the presence of dicoumarol in plants is relatively rare apart from molded sweet clover, being cited only in sweet vernal grass (*Anthoxantum odoratum* L [Poaceae]) (Polya, 2003), therefore the risk of an accidental anticoagulant effect with hemorrhage after ingesting dietary plants rich in coumarins is probably rather low. The structural characteristics (hydroxy groups) that enable dicoumarol to effectively block vitamin K epoxide reductase (VKOR) are not present in the molecule of coumarin. Furthermore, a small scale clinical study with coumarin administered orally to patients with chronic venous insufficiency in doses of 90 mg/day for 6 weeks, failed to demonstrate any effect of coumarin on coagulation parameters (Köstering et al., 1985). Even though other natural coumarins could present anticoagulant effects, there are no sufficient studies to reach a clear conclusion.

### Dermatological Toxicity

Among natural coumarins, several compounds like psoralen, bergapten and xanthotoxin, all belonging to furanocoumarin class, present in large concentrations in celery or limes, caused a limited number of skin phototoxic reactions in humans (Wagstaff, 1991). Thus, several cases of photoallergic reactions were also demonstrated in patients exposed to furanocoumarins from fig, the adverse reaction being confirmed by histopathological examination of patch tests (Bonamonte et al., 2010). However, a typical furanocoumarin intake from food sources is several times below the lowest dose capable of producing phototoxic effects, but the risk of exposure increases in case of inappropriate storage or processing of foods (Guth et al., 2011).

Additionally, a case of severe exacerbation of rosacea induced by cinnamon dietary supplements was reported in a 68-year-old patient but the adverse effect could not be attributed to a specific coumarin present in the chemical composition of the supplement administered with the purpose of lowering glycemia (Campbell et al., 2008). Also, several case reports signaled the development of contact dermatitis caused by cinnamon-flavored toothpaste, chewing gum and mouthwash (Calapai et al., 2014).

Moreover, in case of furanocoumarins, the capacity to form interstrand crosslinks with DNA and to alter DNA transcription may favor the development of skin melanoma, but further research is necessary to ascertain the validity of this hypothesis (Melough and Chun, 2018).

### Reproductive and Developmental Toxicity

Several *in vitro* and *in vivo* models evaluated the reproductive and developmental toxicity of coumarins. A study on zebrafish (*Danio rerio*) embryos showed that coumarin caused malformations of head and tail of zebrafish embryos but the calculated LC50 was 855 μM, suggesting that in humans, under normal therapeutic conditions, teratogenicity could be rather low for coumarin, unlike warfarin (Weigt et al., 2012). Moreover, a recent study investigated the effects of a series of coumarin derivatives on zebrafish embryos, finding no modifications in the developing larvae and no apparent damage to their internal structures (Aspatwar et al., 2020). Additionally, an *in vivo* study showed that oral administration of high doses of coumarin to male and female Wistar rats, prior and during mating phase, produced no adverse effects concerning parental fertility or the development of rat pups (Api et al., 2019). In humans, no data concerning reproductive and developmental toxicity of natural coumarins have been published so far.

### Drug Interactions

Coumarin derivatives are a class of chemically diverse compounds, capable of interfering with the metabolism or the effects of other drugs. Thus, several constituents from grapefruit juice like bergapten, a furanocoumarin derivative, are inhibitors of CYP3A4 liver microsomal enzyme, capable of reducing the metabolism of several associated drugs like calcium channel blockers (nitrendipine) or statins (simvastatin) with the augmentation of their adverse effects. Nevertheless, other chemical compounds like naringenin, a flavanone also present in the chemical composition of grapefruit juice are additionally responsible for this pharmacokinetic interaction (EMA, 2012).

Moreover, a recent study evaluated *in vivo* in rats and rabbits with alloxan-induced diabetes, the pharmacokinetic and pharmacodynamic interactions of cinnamon bark powder and pioglitazone, an oral antidiabetic drug from the class of thiazolidinediones. The results showed that cinnamon was able to inhibit CYP3A4 enzyme activity, increasing AUC of pioglitazone which is metabolized by the same isoform. Additionally, the antidiabetic effect of cinnamon, demonstrated in several studies, could increase the hypoglycemic effect of pioglitazone, an adjustment of the dose being recommended in human patients (Mamindla et al., 2017).

Another individual coumarin molecule, osthole was tested *in vitro* on rat and human liver microsomes regarding the effects on CYP2C11/CYP2C9 enzymes. The results showed a potent CYP2C9 inhibition in human liver microsomes with Ki values between 13.12 and 21.93 µM, also proving an influence of genotype on the pharmacokinetics of osthole. Thus, the presence of CYP2C9*3 allele caused the strongest enzymatic inhibitory activity of osthole in this experimental model (He et al., 2020).

### Regulatory Aspects

A variety of foods and herbs used as nutraceuticals may have a high concentration of natural coumarins, therefore regulatory authorities around the world took legislative actions in order to avoid possible toxicities in the general public.

Initially, in the European Union, a limit of 2 mg/kg coumarin for foods prepared with natural spices and herbs was imposed. Several years later, the European Food Safety Authority (EFSA) recommended a maximum level of 0.5 mg/kg in foods. Based on various animal data, extrapolated to humans, a tolerable daily intake (TDI) of 0.1 mg/kg bw coumarin was calculated (EFSA, 2004). Nowadays, in the European Union, the presence of coumarins in food is regulated by the Decision No 1334/2008 of the European Parliament and Council which states that coumarin cannot be added to food as an additive. However, the Annex III of the document, stipulates that coumarin may be allowed in specific foods prepared with cinnamon as a flavor but with maximum admitted levels (e.g., 50 mg/kg for traditional bakery products and 5 mg/kg for desserts) (European Commission, 2008).

In the USA, coumarin was used as a food flavor until 1954, when its addition to food was banned by the FDA on suspicion of hepatotoxicity (Abraham et al., 2010). Therefore, any food with added coumarin is considered to be “adulterated under the act” being strictly prohibited in the US (Food and Drug Administration, 1999).

In Australia, coumarin itself was authorized for the treatment of lymphedema in 1993, but in 1996 the Australian regulatory authorities suspended the drug due to the apparition of ten cases of hepatotoxicity with two fatalities. Currently, due to the extensive use of coumarin as an ingredient in cosmetic products (sunscreens), the Australian regulatory authorities imposed a limit of maximum 0.001% coumarin in topical cosmetics, which is considered safe, with a maximum estimated exposure of below 0.02 mg/kg (Therapeutic Goods Administration, 2019).

The regulatory aspects concerning the safe use of coumarins in foods or cosmetics are quite variable worldwide. Moreover, some important aspects have not been regulated at all, like the maximum admitted level of coumarin in cinnamon itself. As a consequence, some foods, and spices with a high content of coumarin which can lead to potential toxicity are still used nowadays. The best example is the cheaper cassia cinnamon (*Cinnamomum aromaticum* Nees [Lauraceae]) which often replaces true cinnamon (*Cinnamomum verum* J. Presl [Lauraceae]) as flavor used in bakery products, generating coumarin levels over 50 mg/kg, well above the upper limits set by the regulatory authorities (Yarnell and Abascal, 2009). Further studies aimed at a better understanding of the bioavailability of natural coumarins from foods and cosmetics, but also harmonization measures at international level regarding regulatory aspects are needed, for a safer use of these compounds.

## Conclusion

Natural coumarins present in a variety of foods and herbs are a class of chemically diverse compounds with important biological effects, useful for health promotion and the prevention of various diseases. The most important adverse effects of coumarins are represented by hepatotoxicity favored by the ingestion of large doses and possible genetic polymorphism of CYP2A6 and dermatological phototoxic reactions. A better understanding of the safety profile of coumarins present in nutraceuticals is necessary for a safer use of these valuable natural compounds.
